# A technical note on emerging combination approach involved in the onconanotherapeutics

**DOI:** 10.1080/10717544.2022.2132018

**Published:** 2022-10-13

**Authors:** Mohammad Kashif Iqubal, Harsimran Kaur, Shadab Md, Nabil A. Alhakamy, Ashif Iqubal, Javed Ali, Sanjula Baboota

**Affiliations:** a Product Development Department, Sentiss Research Centre, Sentiss Pharma Pvt Ltd, Gurugram, India; b Department of Pharmaceutics, School of Pharmaceutical Education and Research, Jamia Hamdard, New Delhi, India; c Department of Pharmaceutics, Delhi Pharmaceutical Science and Research University, New Delhi, India; d Department of Pharmaceutics, Faculty of Pharmacy, King Abdulaziz University, Jeddah, Saudi Arabia; e Department of Pharmacology, School of Pharmaceutical Education and Research, Jamia Hamdard, New Delhi, India

**Keywords:** Chemotherapy, nanotechnology, dual-drug combination, immunotherapy, personalized medicine

## Abstract

Cancer is the second cause of mortality worldwide, and the currently available conventional treatment approach is associated with serious side effects and poor clinical outcomes. Based on the outcome of the exploratory preclinical and clinical studies, it was found that therapeutic response increases multiple folds when anticancer drugs are used in combination. However, the conventional combination of anticancer drugs was associated with various limitations such as increased cost of treatment, systemic toxicity, drug resistance, and reduced pharmacokinetic attributes. Hence, attempts were made to formulate nanocarrier fabricated combinatorial drugs (NFCDs) to effectively manage and treat cancer. This approach offers several advantages, such as improved stability, lower drug exposure, targeted drug delivery, low side effects, and improved clinical outcome. Hence, in this review, first time, we have discussed the recent advancement and various types of nano carrier-based combinatorial drug delivery systems in a different type of cancer and highlighted the personalized combinatorial theranostic medicine as a futuristic anticancer treatment approach.

## 1. Introduction

**Figure 1.**
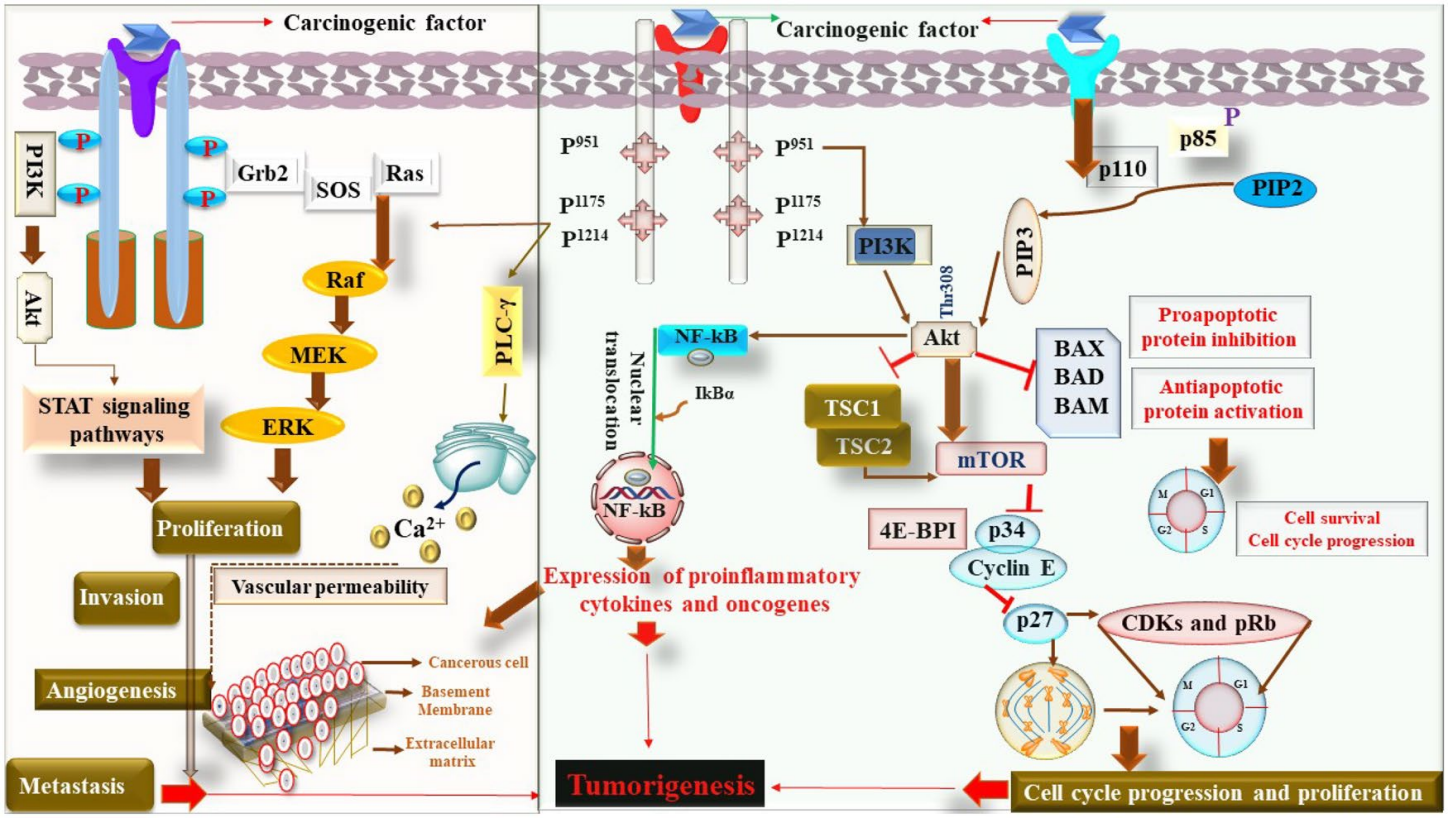
The molecular mechanism of carcinogenesis.

Cancer is one of the leading causes of mortality and morbidity worldwide. More than 10 million deaths have been recorded due to different types of cancer. Lung cancer (18%), followed by a colon (9.4%) and liver (8.3%), cancer is the leading cause of death. Considering the incidence of cancer, breast cancer ranks first (11.7%), followed by the lung (11.4%), and colon (10%). Moreover, as per the WHO report, in China, a maximum number of deaths have been reported, followed by India and the USA, whereas the incidence of cancer is highest in China, followed by the USA and India, as shown in Figure 1 (Siegel et al., 2022). Considering the etiology of cancer, it is multifactorial in origin but begins from the alteration in DNA. No doubt, mutations are primarily responsible for various types of cancer. Still, apart from the role of mutation and mutagens, various other oncogenic factors such as radiation, chemical exposures, stress, exposure of drugs, etc., are also equally responsible (Maiorino et al., 2022). Recent studies have shown that epigenetic pathways that regulate the expression of various genes also play a pivotal role in the event of carcinogenesis. Studies have further shown abnormal methylation, such as hypo or hypermethylation, and histone deacetylase activity in the carcinogenesis pathogenesis (Fardi et al., 2018). Hence, the cumulative effect of mutational factors and abnormal epigenetic mechanisms is responsible for the emergence of the malignant phenotype ‘malignant transformation’. Moreover, based on the exploratory studies, it was found that multiple pathways are accountable for the transformation of a normal cell into a malignant cell. No doubt, tumorigenesis begins clonally with a single cell and undergoes a neoplastic transformation. Hence, the identification of the clonal cells is a pivotal indicator for the initiation of tumorigenesis (Nichenametla et al., 2006).

It is also important to understand that in response to persistent injury, normal tissue responds by the loss of proliferation mechanism, which indeed is regulated by the specific receptors, signaling molecules, enzymes, and other biochemical machinery (Tuli et al., 2021). In normal physiological conditions, the proliferative process is a highly controlled and regulated process that retains the homeostatic functions. In contrast, uncontrolled proliferation occurs during carcinogenesis, leading to tumor initiation, progression, invasion, and metastasis (Iqubal et al., 2021b). Moreover, in the event of tumorigenesis, oncogenes play a pivotal role. These oncogenes have been divided into five categories: growth factors, their receptors, signal transducers, transcription factors, and various cyclins (Croce, 2008). Some commonly identified growth factors and their receptors are vascular endothelial growth factor-receptor (VEGF/VEGF-R), epidermal growth factor-receptor (EGF/EGFR), Erb B, etc., whereas some of the commonly identified prooncogenic intracellular signal transducers are rapidly accelerated fibrosarcoma (RAF), rat sarcoma virus (RAS), etc. Transcription factors involved in oncogenesis primarily regulate gene expression via binding to the specific site of DNA. MYC, JUN, and FOS are some of the commonly studied transcriptional factors. Cyclins, on the other hand, are proteins that modulate the cell cycle. These cyclins play their role at specific checkpoints of cell cycles, interacting with various enzymes known as cyclin-dependent kinases (CDKs) and enabling the progression of the cell cycle. Exploratory studies have found various CDKs, such as cyclin D1 and get dysregulated and participate in the event of tumorigenesis (Iqubal et al., 2020).

It is further important to understand that structural alteration in genes also transforms them into oncogenes. The structural damage mainly occurs because of mutation of nucleotides or due to chromosomal translational events manifested by the chemicals or radiation (Jones & Baylin, 2007). The best example can be seen in the case of RAS proteins that exist in three forms, K-RAS, N-RAS, and H-RAS and act as a GTPase that regulates the phosphorylation of GTP, leading to cell proliferation and cell survival. In the case of their mutation, Ras members bind to the receptors of growth factors, undergoes dimerization, autophosphorylation and conformational changes leading to uncontrolled cell division and survival of tumor cells (Porter et al., 2016).

Doubtless, tumor cell survival is a major clinical concern for clinicians, but at the same time, angiogenesis and metastasis is another important concern that considerably affects the clinical outcomes. Vascularization is important for the growth of tumor cells, and here the role of VEGF and FGF is decisive (Kawada & Taketo, 2011). Apart from the role of growth factors, matrix metalloproteinases (MMPs), collagens nuclear factor kappa B (NF-kB), and other cytokines also play a pathological role in the event of angiogenesis and hence the progression of tumor cells. During the course of angiogenesis, continuous leakage of macromolecules and deposition of fibrins act as a tool for tumor stroma formation and their migration that eventually leads to tumor metastasis (Iqubal et al., 2020).

Henceforth, it can be concluded that the incidence and prevalence of cancers are increasing exponentially and affect the lives of patients considerably. No doubt, with the time and advancement in the cellular and molecular techniques, detailed cellular and sub-cellular pathology of cancer have been explored and various newer drugs have been developed (Iqubal et al., 2020). Despite numerous advancements in treatment modality using chemotherapy, radiotherapy, surgical intervention, and immunotherapy, cancer patients’ survival rate and quality of life have not improved considerably (Ayoub, 2021). Thus, attempts were made to use a combination of chemotherapy and immunotherapy to improve the quality of life of chemotherapeutically treated patients (Al-Lazikani et al., 2012). Hence, in the present review, we first discussed the combinational therapy of conventional drugs, their limitations, and the pharmacotherapeutic role of various onconanotherapeutics used in various types of cancers.

## 2. Oncotherapeutics for the management and treatment of cancer

The concept of combination therapy was first implemented by Emil et al., in 1965 in one of a pediatric patient diagnosed with acute leukemia, and the outcome was successful (Frei et al., 1965). After the success of this combination therapy, the entire journey of treatment and management of changed considerably. More and more emphasis were put on the rationale exploration of various combination therapy with the aim of targeting multiple pathways to generate favorable clinical outcome (Ismail et al., 2020). In this journey, genomics, oncogenomics, proteomics, and transcriptomics played a significant role in the identification of various molecular targets for the combinational anticancer drugs (Falzone et al., 2018). Based on the published evidence and our previous work, we concluded that the combinational therapeutic approach offers several benefits over single therapy (Md et al., 2020). Firstly, combinational therapy significantly improves the clinical outcome, exhibit superior therapeutic effect as compared to the single drugs. Combinational drugs effectively overcome the clonal heterogenicity, and reduced the toxicity as a comparatively lower amount of drug is used. Moreover, combinational therapy significantly reduces drug resistance and increases patients’ survival rate (Ayoub, 2021).

No doubt, combinational therapy offers several advantages over single drug therapy, but still concern exists. One of the major issues with this approach is drug–drug interaction and unpredictable pharmacokinetic profile that eventually influence the desired clinical outcome. Additionally, when a lower amount of drug is used to avoid toxicity, sometime results in a suboptimal therapeutic response and also the predicted synergistic effect is inconclusive (Chou, 2010). It is further important to bring in notice that most of the combinational drug used among the cancer patients were primarily based on the empirical clinical experimental settings and unfortunately, the mechanistic approach and the rationale for the combination was lacking (Palmer et al., 2019). Thus, to overcome these issues, Narayan et al. have used the typical approach known as the ‘drug atlas’, where novel and rationale drug combinations with their synergistic effects were explored. In the approach of ‘drug atlas’ specific pathways and processes were identified against which tumor cells can be attacked and killed by combination of drugs (Narayan et al., 2020). ‘Restrictive combination’ is another approach where a clear difference is observed between cancerous and normal cells upon application of dual drugs (Blagosklonny, 2008). More recently, Tolcher et al. have designed and developed ‘CombiPlex’ technology where the probability of possible anticancer activity and a synergistic effect was evaluated (Tolcher & Mayer, 2018).

Colombo et al. have evaluated the anticancer effect of birinapant (apoptosis inhibitor) and ralimetinib (p38 inhibitor) in the liver kinase B1 deleted clone and RAS muted cell lines and the outcome showed restoration of kinase activity (Colombo et al., 2020). In another study by Mortensen et al., the combination of onalespib and cisplatin significantly increased the antiproliferative, pro apoptotic, and antimigratory effect (Mortensen et al., 2020). In one of the interesting findings, Shi et al. showed that when regorafenib and ABC294640 (SphK2 inhibitor) were used in liver cancer, regorafenib resistance was significantly reduced and marked improvement in the anticancer effect was observed, as compared to the individual drugs (Shi et al., 2020). Drug resistance is one of the major concerns for the oncologist world-wide, and the use of a single drug is extensively reported to exhibit drug resistance (Bukowska et al., 2015). Studies have shown that overexpressed ATP-binding cassette (ABC) efflux transporters are associated with resistance and considering this, Yang et al. have explored the effect of sitravatinib (a tyrosine kinase inhibitor) as a blocker of ABC efflux transported and exhibited a significant anticancer effect (Bukowska et al., 2015; Ju Yang et al., 2016).

Drug repurposing is among the emerging approaches for the use of already FDA-approved drugs for the treatment and management of various types of cancer. In one of the interesting studies, Hsu et al. explored the synergistic effect of sildenafil (phosphodiesterase inhibitor) and vincristine (calcium channel blocker) against castration-resistant prostate cancer (CRPC). The outcome of the study showed that sildenafil exhibited a synergistic effect with vincristine and induced cell cycle arrest (Hsu et al., 2020). Hence, the use of combination therapy in the treatment and management of cancer has become standard practice. Henceforth, many FDA-approved drugs and their combinations are routinely used. Despite, this targeted mechanism of action, low solubility, low bioavailability and reduced therapeutic outcome are major limitations (Table 1).

**Table 1.** Oncotherapeutics for the management and treatment of cancer (The ASCO Post Staff, 2020).

Drug 1	Drug 2	Type of cancer
Ipilimumab	Nivolumab	Advanced kidney cancer Advanced colorectal cancer Non-small-cell lung cancer (NSCLC)
Cisplatin	Navelbine	NSCLC
Doxorubicin (DOX)	Cyclophosphamide	Breast cancer
DOX, vinblastine	Bleomycin, dacarbazine	Breast cancer
5-FU	Axitinib	Breast cancer
5-FU	Alpelisib	Breast cancer
5-FU	Afatinib	Colon cancer
5-FU	Crizotinib	Pancreatic cancer
Carfilzomib	Daratumumab, dexamethasone	Refractory multiple myeloma
Daratumumab	Hyaluronidase-fihj	Relapsed/refractory multiple myeloma
Atezolizumab	Cobimetinib and vemurafenib	Metastatic melanoma
Atezolizumab	Bevacizumab	Unresectable or metastatic hepatocellular carcinoma
Decitabine	Cedazuridine	Myelodysplastic syndrome
Pertuzumab	Trastuzumab, and hyaluronidase	Multiple myeloma
Tucatinib	Trastuzumab and capecitabine	Her2-positive breast cancer
Neratinib	Capecitabine	Her2-positive breast cancer
Ramucirumab	Erlotinib	Metastatic NSCLC
Olaparib	Bevacizumab	Advanced epithelial ovarian, fallopian tube, or primary peritoneal cancer
Ibrutinib	Rituximab	Chronic lymphocytic leukemia
Encorafenib	Cetuximab	Metastatic colorectal cancer
Durvalumab	Etoposide	Small cell lung cancer
Isatuximab-irfc	Pomalidomide and dexamethasone	Multiple myeloma

5-FU: 5-Florouracil; DOX: Doxorubicin.

## 3. Onconanotherapeutics

**Figure 2.**
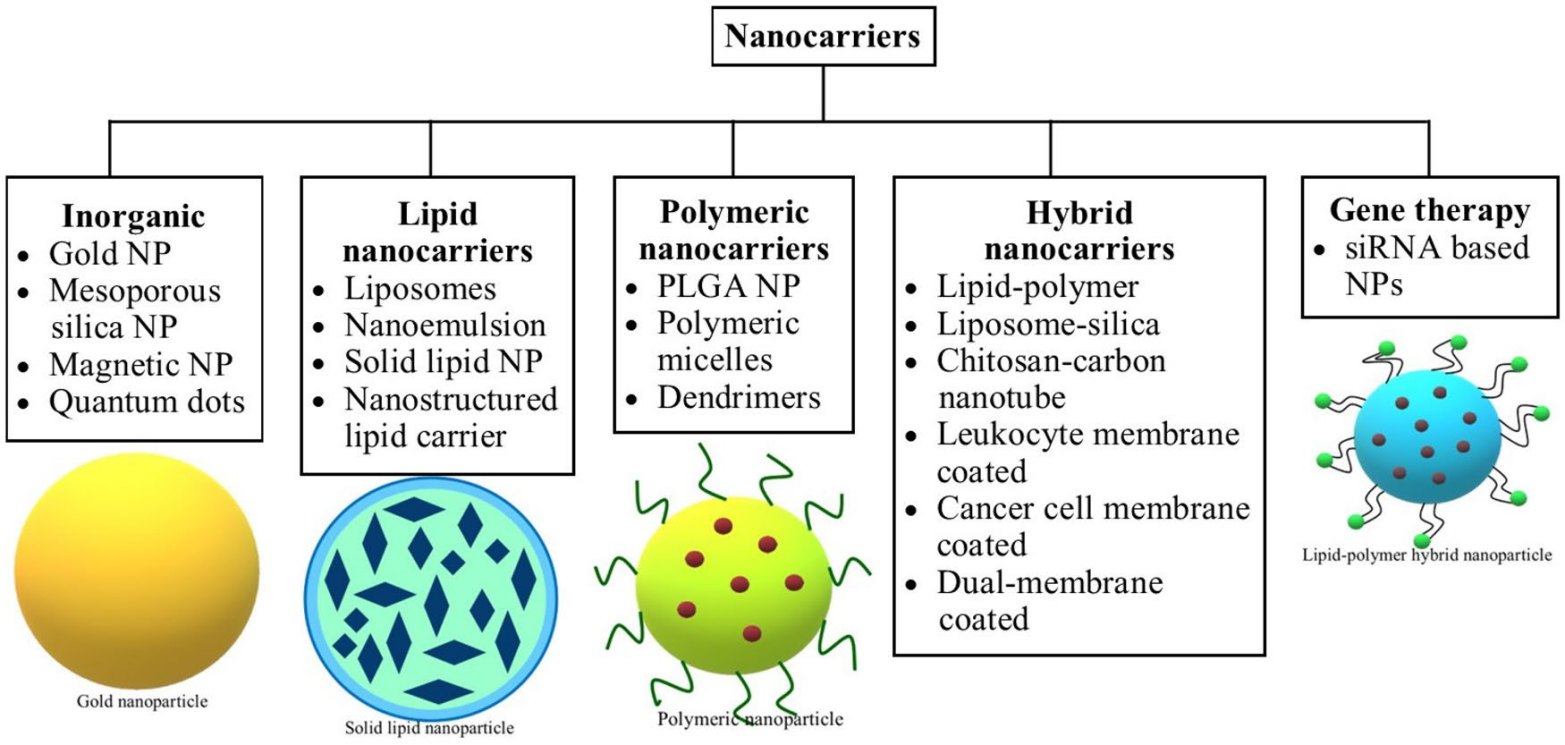
Brief classification of nanocarriers.

Nanotechnology has been progressively utilized since the past few years for therapeutic purposes, including applications for diagnosis, treatment, and tumor targeting in a safe and efficacious manner. The nanocarriers used for therapeutic benefits account for specific sizes, shapes, and surficial properties owing to their influence on nanocarrier-based drug delivery efficiency and accordingly provide improved therapeutic value. With reference to the size, nanocarriers within diametric sizes ranging from 10 to 100 nm are generally characterized as suitable candidates for cancer therapeutics because they can deliver drugs effectively along with achieving enhanced permeability and retention (EPR) effect. Nanocarrier-based drug delivery systems have exhibited propitious merits in cancer treatment, namely good pharmacokinetics, precise targeting of tumor cells, reduced incidence of side effects, and drug resistance (Dadwal et al., 2018; Palazzolo et al., 2018). Nanocarriers in cancer management are usually selected based on their characteristics and the pathophysiology of the tumors. Several types of nanocarriers, such as protein-based nanoparticles (NPs), silica-based nanocarriers, liposomes, solid lipid nanocarriers, etc., have been developed (Yoon et al., 2020), and their brief classification is presented in Figure 2. Mechanically, the anti-carcinogenic effect of nanocarriers involves targeting malignant cells through their carrier effect and the positioning impact of the targeting moiety after being absorbed, followed by the release of drugs into malignant cells, as a measure to induce cell death. The drugs entrapped within the nanocarriers include traditional chemotherapeutic agents and nucleic acids, indicating that they can play a crucial part in both gene therapy and chemotherapy. In the bargain, nanocarriers offer a platform for some poorly soluble drugs, which can aid in their encapsulation and deliver the drugs into the circulatory environment (Yao et al., 2020). As a consequence of the size, surface properties, and function of nanocarriers in enhancing permeability and retention, it can increase the half-life of drugs and improve their availability in malignant cells (Gavas et al., 2021; Kalyane et al., 2019). In the interim, the targeting approach shields normal cells from the unwanted effects of drug molecules and helps alleviate the adverse effects of anticancer therapy. For example, DOX-loaded polyethylene glycol (PEG) or PEGylated liposomes impart reduced cardiotoxicity in comparison to free DOX (O’Brien et al., 2004). Furthermore, various studies have shown the application of nanocarriers in immunotherapy for cancer (Riley & Day, 2017; Yoon et al., 2018), and this approach of the delivery system is executed with the belief of enhancing immunotherapy besides reversing the tumor immunosuppressive microenvironment (Yao et al., 2020).

In the late years, a rising number of nanocarrier-based drug delivery systems have been commercialized or entered the stage of clinical trials. The phase I clinical trial that utilized a targeted NP-based system to deliver small interfering RNA (siRNA) in patients with solid tumors was conducted in 2010 (Davis et al., 2010). Another clinical study revealed a promising tumor treatment competency of actively targeted polymeric NPs containing the chemotherapeutic docetaxel in comparison to a solvent-based docetaxel formulation (Hrkach et al., 2012). The formulation of hybrid nanocarriers has paved the way in the area of nanocarrier-based drug delivery systems. Hybrid nanocarriers offer combined benefits of different nanocarriers; as a result, enhancing the stability and functionality of every delivery system (Yao et al., 2020). Over and above, nanocarriers have unveiled certain advantages with respect to antitumor multidrug resistance (MDR), as a consequence of offering podium for combination drug therapy as well as hindering the biz of some mechanisms of drug resistance, like efflux transporters on cell membranes (W. Li et al., 2016). Presently, nanocarrier-based therapy has been reported to have prospects of overcoming MDR in several types of cancers, including prostate cancer (J. Zhang et al., 2019), ovarian cancer (H. Wang et al., 2018), and breast cancer (Alimoradi et al., 2018). Ergo, nanocarriers in the field of medicine have laid the foundations for cancer treatment, and combining these two fields demands more in-depth research.

## 4. Combination based onconanotherapeutics

**Figure 3.**
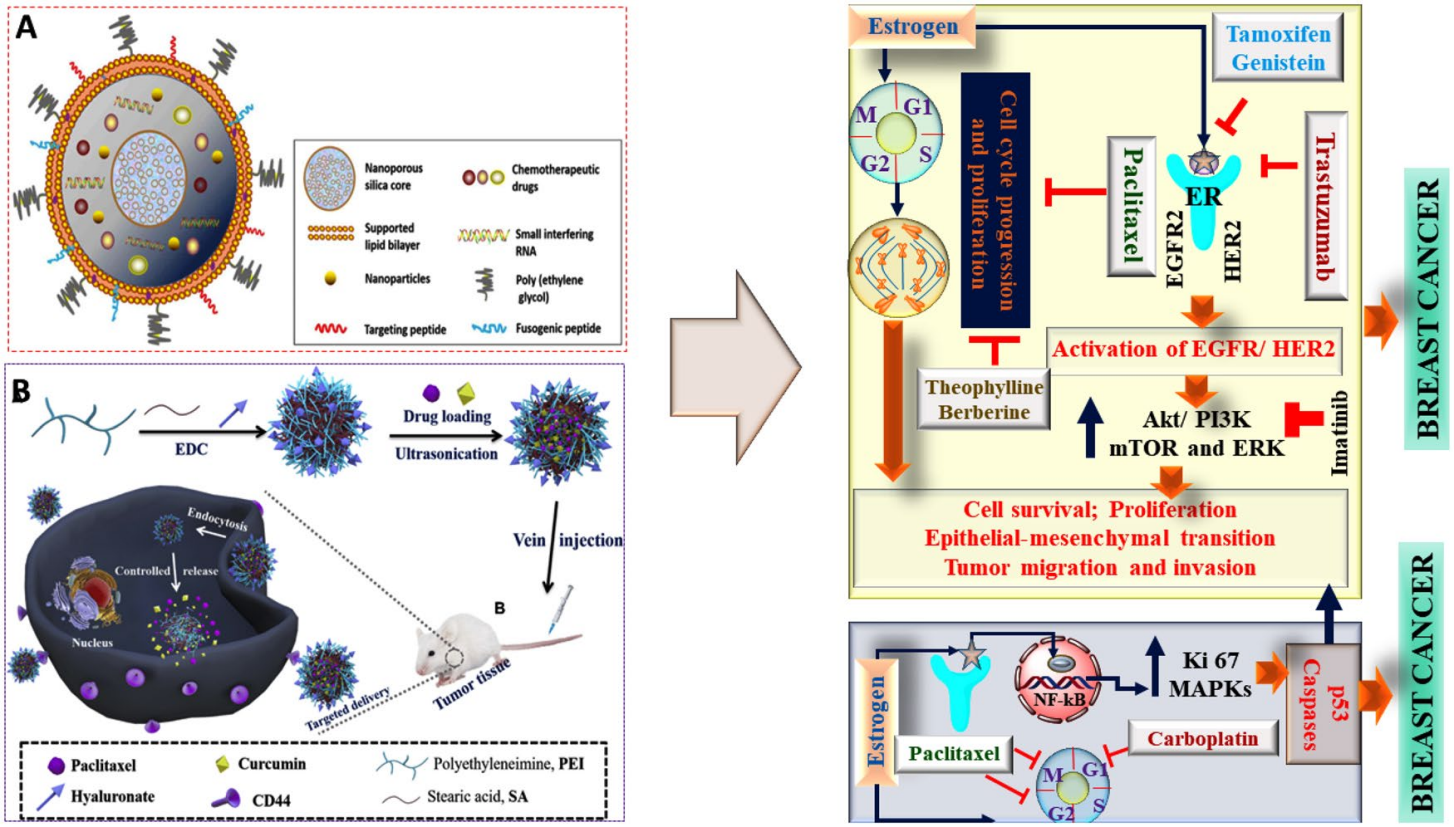
The classical example of nanocarrier based combinational drug delivery in oncotherapy. (A) and (B) showing different types of architects of noncarriers used and mechanism of combinational drug delivery as potent anticancer therapy (Li et al., 2017; Zhao et al., 2019).

As evident from numerous published evidences, various attempts were made by the researchers to manage and treat cancers. Among various treatment modalities, chemotherapy has remained the primary pharmacotherapeutic approach, but instead of many advances, there exists multiple for this approach (Papac, 2010). Previously and also currently, in general, single anticancer drugs were used that specifically target the single cancer signaling pathways. This approach was found to be positively correlated with the drug resistance, side effects, and altered pharmacokinetic attributes (Goodman & Wintrobe, 1946). Hence, attempts were made to use a combination of two or more drug, but again, a major issue of the unpredicted pharmacokinetic and pharmacodynamic profile due to the difference in dosing time were found (Kent & Huber, 1999). Hence, to overcome this issue, a combination based nanotechnological approach was used where different types of nanocarriers were used for the safe and effective drug delivery (Fellmann et al., 2017). A significant advantage of using combination based nanocarrier is the encapsulation of multiple drugs with an increased load capacity that effectively deliver the drug at the targeted site at a pre-determined rate. Another advantage of using nanocarriers for combinational drug delivery is the use of a lower drug dose that results in better safety profile and the possibility of avoiding drug resistance. Moreover, the use of a nanocarrier for the dual drug delivery also helps in bypassing the drug uptake via endocytosis and exhibits superior bioavailability (Yasmeen et al., 2021). In conclusion, the nanoformulated combinatorial drugs (NFCDs) delivery system offers several advantages, such as synergistic effect, minimal drug resistance, controlled drug release, and superior anticancer effect with an improved safety profile as shown in Figure 3.

### 4.1. Colorectal cancer

A recent study involved the combined efficacy of two anticancer drugs, namely SN38 (an active form of irinotecan) and salinomycin (Sal), an ionophore, by encapsulating them into lipid nanocapsules (LNC). The selective combination was proposed to reduce the systemic toxicity and enhance the stability. The clonogenicity assay performed on HCT116 cell lines revealed the IC_50_ values for SN38 as 0.5 ng/ml and for Sal as 2.4 µg/ml. Activation of GLS2 (essential ferroptosis activator), MT1 gene and upregulation of CHAC1 and PTGS2 (prototypic markers of ferroptosis) was observed, as a result of ferroptosis activation, mediated by Sal, which confirmed its anticarcinogenic effect. The combined and simultaneous treatment of both the drugs along with the IC_50_ concentrations exhibits a synergistic effect at combination index (CI) < 0. The resultant formulation, i.e., the LNC were in the size range of 50 nm. The combined drugs showed a great efficiency to target CRCs in 3D structures, independent of cell heterogeneity and plasticity. The overall results proved that SN38 and Sal together are a promising solution for treating CRCs (Tsakiris et al., 2020).

In another experiment, emulsomes were synthesized by incorporating curcumin and piperine to achieve a synergistic anticarcinogenic effect. The average diameter of curcumin loaded emulsomes was observed to be 184.21 nm, whereas piperine linked emulsomes had a larger average size of 248.76 nm. When administered individually, piperine both in its free and emulsome forms did not exhibit any remarkable change on HCT116 cell viability, *in vitro* but as an adjuvant it showed a significant enhancement in curcumin’s action. Piperine and curcumin-loaded emulsomes, combined in concentrations of 7 µm and 25 µm, respectively, acted as a suitable therapy for inducing cancer cell death. G2/M arrest in the HCT116 cell line and induction of apoptotic response further confirmed the anticancer properties of the compounds selected. A four-fold increase in caspase 3 level was obtained with curcumin-emulsomes while a six-fold increase was reported with piperine-emulsomes. Hence, the overall studies indicate the antitumor activity of the compounds which proves them as a suitable approach for further *in vivo* studies (Bolat et al., 2020).

In one of the studies, PEG-linked poly(caprolactone) block copolymer (PCL) was utilized for the co-loading of 5-FU and plasmid encoding EGFP. The average hydrodynamic size of empty nanocarrriers and 5-FU-loaded nanocarriers was reported to be around 110 nm, whereas the DNA-trapped DNA-loaded nanocarriers and DNA and 5-FU co-loaded nanocarriers showed size of about 145 nm. The PDI was found to be below 0.2. The drug and gene entrapment efficiency of 5-FU was observed to be around 80% and 90%, respectively. A dose-dependent cytotoxicity was seen in the 5-FU containing groups. The inhibition capacity in colon cancer cells was stronger with 5-FU-loaded nanocarriers as opposed to free 5-FU. The tumor volume of 5-FU-loaded nanocarriers and DNA and 5-FU co-loaded nanocarriers was significantly reduced, which resulted in the suppression of tumor growth. The drug and gene co-loaded nanocarriers exhibited antitumor properties to a better extent and efficient gene delivery was attained at the target site (Z. Wang et al., 2018).

Chimeric antigen receptor modifiedT cells are a new class of candidates for the treatment of carcinoma. Zhang et al. utilized these anticancer agents in a treatment model for colorectal cancer. The study involved the formation of chimeric antigen receptor (CAR)belonging to second-generation, targeting epithelial cell adhesion molecule and a combined approach of CAR specific natural killer (NK) cells along with regorafenib was employed to exploit their synergistic potential. The escalating response in the *in vitro* release profile of cytokines in NK-92 cell lines linked to CAR, is attributed to the recognition ability and activation mechanism mediated by EpCAM, i.e., positive colon cancer cells. A remarkable reduction in the HCT-8 tumor growth was observed by the co-administration of regorafenib and CAR associated NK-92 cells, in contrast to the dose of plain moieties. This further highlights the synergistic anticarcinogenic capacity of the combination to target CRCs (Q. Zhang et al., 2019).

### 4.2. Skin cancer

Dacarbazine, known as a DNA alkylating agent, possesses potent chemotherapeutic activity. However, associated with limitations such as short half-life and poor aqueous solubility, it offers reduced efficacy. To overcome these barriers, Li and Han selected all-trans-retinoic acid, an anticarcinogenic compound to promote co-delivery along with dacarbazine that would result in a synergistic effect as compared with the individual compound. The research was conducted to target melanoma malignancies by formulating a combination of dacarbazine and all trans-retinoic acid-loaded lipid nanoformulations, thereby resulting in enhanced antitumor efficacy, attributed to the synergistic action. The particle size of nanoformulation was reported to be 121.5 ± 1.65 nm with zeta potential of −23.5 ± 0.85 mV and PDI of 0.134. The particle size of optimized formulation was 138.2 ± 1.28 nm with zeta potential of −25.4 ± 0.58 mV and PDI of 0.159. Liposomal carriers exhibited significant anticarcinogenic toward the melanoma cancer cell death. In addition, inhibition of cell cycle progression and greater apoptosis mechanism was seen in the B16F10 cells after treatment with the selected formulation, which indicates excellent antitumor efficacy of the combinational approach. The results prove that the lipid complex suppresses melanoma cell proliferation, induces significant apoptosis and restricts cell cycle progression and migrations, which concludes it as a suitable candidate for melanoma malignancies (Li & Han, 2020).

To overcome the hindrance associated with dermal penetration of therapeutic candidates, nanoencapsulated dabrafenib (Db) and trametinib (Tb) were formulated using silica NPs linked to phthalocyanine (Pc), as a measure to prolong the circulation time and induce synergistic benefits via co-delivery approach. Anthracenediylbis(methylene)dimalonic acid (ABDA) was introduced as a trapping agent which differentiated the quenching efficiency of different formulations, suggesting that the amount of phthalocyanine and tetramethyl orthosilicate (TMOS) had a great significance in inducing appropriate response. The PDI, diametric size, and zeta potential figures were reported to be between 0.157 to 0.165, 29 to 39 nm and −20.5 to −22.1 mV, respectively. The administration of formulation in addition to the photodynamic therapy, exhibited a major apoptotic response, as evident from the intensified red fluorescence. The killing efficiency was more prominent with the combined treatment rather than single moieties. Thus, from the findings, it can be concluded that Db-Tb-Pc loaded silica NP contribute to the therapeutic regimen in synergy with photodynamic treatment (PDT) treatment (Tham et al., 2018).

Long et al. reported the combined effect of photothermal (PTT) and PDT, to destruct the tumor microenvironment while potentiating drug release from manganese dioxide and indocyanine green loaded derivative, incorporated in to the surface of bovine serum albumin (BSA) molecules for exploiting the anticarcinogenic properties. The fluorescence pattern of optimized nanocomplex was further studied using singlet oxygen sensor green (SOSG) medium, which resulted in generation of singlet oxygen species as an indicator of promising photodynamic effect. The cell destruction ability was confirmed at higher dose of formulation adjuvant to PTT and PDT therapies. The responses calculated depicted a huge difference in the cell death. Responses to the laser radiations can be related to the elevation in temperature and production of singlet oxygen. An impressive tumor inhibition was seen after subjecting mice to IV administration of the formulation along with laser treatment, which highlights the proliferative action of nanoconjugate prepared (Wen et al., 2020).

In another study, celecoxib and plumbagin were combined together to formulate nano liposomes conjugate, which provided synergistic action to induce cell death. The mechanisms included inhibition of cyclooxygenase-2 (COX-2) and signal transducer and activator of transcription 3 (STAT3) pathway, which are the key targets in melanoma. The combined cytotoxic potential was confirmed by the CI values that were below 0.9 at 10:1 ratio of Cele and Plum. The normal cells did not exhibit any cytotoxic response while the melanoma cells underwent apoptosis with the dual-drug complex. Celeplum-777 induced a decline in cellular growth (62% in aqueous medium and 72% in saline base). The complementary enhancement in protein expression leads to successful inhibition in COX-2 pathway. Also reduced cyclin levels in melanoma and UACC 903 cell lines, restricted the proliferative mechanism and cell-arrest events. In nutshell, the unique model contributed to suppress tumor growth, thereby targeting crucial markers responsible for cell survival (Gowda et al., 2017).

PDT offers advantages in the field of skin ailments. Using this approach, the study by Nasr et al. involved comparison of the delivery profiles of ethosomal-chlorophyll derivative complex and chitosan nanocompounds loaded with layer of lipids, further linked to ferrous chlorophyll moieties. The skin retention capacity was reported to be 28% for lipidic chitosan conjugate, whereas 9% retention was observed for nanocarriers of ferrous derivative of chlorophyll in 24 hours time period. The optimized formulations administered to squamous cells were successful in inducing a declined response in the viable cells in synergy with the PDT, whereas no comparable difference was seen in the absence of laser therapy. The destruction of the membranous periphery of A431 spheroids subjected to lipidic carrier was a distinct feature to that of ethosomal conjugate, that caused insignificant effects to the tumor layer. Additionally, an escalating cytotoxic mechanism was observed in the group treated with lipid NPs, in contrast to the ethosome treated. Both nanoformulations acted in a synergistic way with PDT that paves a preferential selection to squamous cell carcinoma therapy (Nasr et al., 2019).

In another study, Iqubal et al. reported a synergistic effect of 5-FU when given with resveratrol via nanostructured lipid carrier (NLC) against skin cancer. In this study, the first optimized combinatorial NLC of 5-FU and resveratrol was selected, which had a particle size of 178.97 ± 2.54 nm and applied topically on diseased area. The therapeutic efficacy of NLC was evaluated by both *in vitro* and *in vivo* studies through estimation of histopathology, ultrastructural analysis, histoimmunology, etc., which exhibited significant (*p* < .05) result for nanoformulation as compared to conventional formulation (Iqubal et al., 2021a, 2021b).

### 4.3. Breast cancer

To improve the poor therapeutic effectiveness due to the limited bioavailability and low water solubility of paclitaxel (PTX), it was nanoformulated with dexamethasone (DEX). Hence, the study investigated the chemosensitizing role of combined treatment of PTX loaded PLGA NPs with DEX. Cellular proliferation was inhibited by PTX-NPs with IC_50_ value reported as 6.67 μg/ml. The results revealed that MCF-7 cells showed decreased survival rate which confirms induction of dose dependent decrease in cell viability due to action of PTX. PTX-NPs showed a three-fold decrease in the IC_50_ value. Major apoptotic section in MCF-7 cells was observed by PTX or PTX-NPs. The co-treatment of DEX/PTX in breast cancer cells exhibited the carcinogenic potential of the nanoformulated PTX (Diab et al., 2020).

Doxorubicin (DOX), mostly preferred chemotherapeutic agent is used for treating breast cancer. Despite its antitumor and cytotoxic potential, there are certain factors which limit its therapeutic efficacy. Hence, to overcome this difficulty, DOX is combined with siRNA nanodrug, which is responsible for inhibiting the antiapoptotic gene birc5/survivin. In a study performed by Ghosh et al., a therapeutic combination was applied to arrest tumor growth, which involved low-dose DOX and an antisurvivin siRNA nanodrug (MN-siBIRC5). The effect was examined in murine xenograft models of breast (triple negative) cancer. *In vitro* cell proliferation assay in breast cancer cell line BT-20 was carried out which revealed that DOX showed successful inhibition in cell proliferation at an IC_50_ value below 0.7 µm. After the cells received treatment with DOX in addition to MN-siBIRC5, an apoptotic reaction was observed while the reverse sequential combination abrogated the benefit of the selected drug combination, which indicates that sequence is an important factor that can result in maximum tumor growth inhibition (Ghosh et al., 2014).

Certain cytotoxic drugs pose variable obstructions in the treatment pathway of cancer, which include less bioavailability and toxic events. Ding and the group worked on research that involved triple therapies as a treatment regimen for breast cancer, consisting of a combination of DOX, PTX and siRNA-based survivin, collectively loaded into the NP matrix. The combined anticancerous drugs were employed for their synergistic efficacy *via* triple mechanisms. A photothermal candidate, termed as PDA was projected onto the surface of nanocarrier-DPS complex. The results for photothermal studies of optimized formulation revealed a remarkable destruction of cancerous cells, with increase in temperature and higher loading of polymer linked to dopamine, which highlights the reason that photothermal efficiency is directly dependent on the amount of polymer dopamine conjugate. The antitumor potential explored by triple mechanism reported a rising proportion of cells being destroyed by the applied therapy, which is attributed to the synergistic response of chemical, photothermal, and gene delivery. The capability of such targeted nanocomposites in effectively producing a chemosensitive property suggests a promising treatment option for triple negative breast cancer (Ding et al., 2017).

The escalating issues offered by drug resistance that happen due to hyper expression of P-glycoprotein, occurs in a major proportion of breast cancer. To mitigate this difficulty, an appropriate strategy to regulate the over-expression of P-gp is the need of the hour. In one of the studies, an assembly incorporated BioPerine, isolated from piperine (active constituent of black pepper) into Poly lactic acid. The chitosan-PEG coated BioPerine-PLA nanocarriers exhibited the release of BioPerine in a delayed manner in neutral environment, whereas the release was accelerated in a relatively basic environment, which indicates its dependability on the pH, for its release mechanism. This could point out to the variations in solubility or the differing dissolution rate. The determination of reactive oxygen species level was carried out on MDA-MB 453 cells, which when subjected to the optimized formulation alone, reported a little rise in ROS levels, but in addition to DOX, showed an effective increase in ROS levels, which further demonstrates the pH-dependent profile of ROS intensity. The co-delivered drugs proclaim successful inhibition of tumor cells through sustained release activity in suitable carrier, which provides an evidential support to target drug-resistance breast cancers (Pillai et al., 2020).

A combined nano-therapeutic system was developed by Zafar et al., which involved simultaneous loading of a frontline agent, docetaxel and an active constituent of *Nigella sativa*, thymoquinone (THQ), incorporating together into LNC. The resultant conjugate comprised of triglyceride core and PEG attached to DSPE as the shell. The PEG-DSPE complex is in turn loaded with a vitamin E derivative, referred to as TPGS. The elevated lipid concentrations resulted in a higher particle size, owing to the viscosity parameter, which limits the diffusion of the solvent. The entrapment efficiency and drug loading values of docetaxel and THQ of the optimized formulation were reported to be around 86.79% and 95.17%, respectively, whereas the drug loading percentage was observed to be approximately 1.19% and 2.61%, respectively. The rapid release of free drug from the complex at the beginning, might be associated with the presence of moieties at the nanocapsule surface, followed by a prolonged release rate, which further points to the gradual apoptotic effect. The effective scavenging potential of free drugs was measured using the 2,2-diphenyl-1-picryl-hydrazyl-hydrate (DPPH) assay, which reported significant antioxidant efficacy of THQ, individually as well as in combined form with docetaxel. Such consequence represents no major change in the functional stability of the molecule in presence of DOX. Hence, the proposed nano conjugated system provides a promising chemotherapeutic effect of the synergistic combination of DOX and THQ to tackle breast cancer (Zafar et al., 2020).

### 4.4. Thyroid cancer

Sorafenib (SOR) is an effective antitumor candidate, but due to an increased incidence of adverse events, it has limited applicability. All-trans retinoic acid (ATRA), an intermediate metabolite of vitamin A, is another compound responsible for inhibiting cell proliferation and metastasis and promoting cell differentiation and apoptosis. Li and group developed a formulation by incorporating SOR and all-trans retinoic acid into PEG linked PLGA polymer micelles in order to enhance the antitumor effect of SOR and thereby reduce its side effects. *In vitro* release studies showed slow and sustained drug release. The SOR release from PM/SOR and PM/(SOR + ATRA) was reported to be 61.3% and 62.4% while ATRA released from PM/ATRA and PM/(SOR + ATRA) was observed to be 56.3% and 63.9%, respectively. MTT assay was performed to evaluate the synergistic anticarcinogenic activity of SOR and ATRA on FTC-133 cells. 18.0 mmol L^−1^ SOR and 70.0 mmol L^−1^ ATRA when combined, proved to be an optimum concentration to illicit a desired synergistic antitumor effect. Tumor necrosis was seen in the group that received treatment with PM/(SOR + ATRA). Redifferentiation of thyroid cancer cells was determined by examining the NIS and Tg levels via immunofluorescence staining, which resulted in higher levels. The overall conclusion suggests that combined delivery of SOR and ATRA is a suitable therapeutic strategy to induce apoptosis, promote cell differentiation and suppress cell proliferation of FTC-133 thyroid cancer (Li et al., 2020).

NF-κB signaling plays a crucial role in cell proliferation, angiogenesis, survival, metastasis, invasion, and drug resistance. This study was approached in order to analyze the efficacy of NF-κB pathway inhibition in combination with docetaxel along with ionizing radiation in thyroid cancer cells. The outcomes showed a limited synergy when both docetaxel and ionizing radiation were used by either genetic or pharmacological pathway. Docetaxel and bortezomib, collectively led to a decreased *in vitro* invasion in only 8505C cells. SAHA, an effective candidate was found capable to reduce cell growth but the result is only limited to BCPAP cells. Hence, it was concluded that the NF-κB pathway inhibitors cannot be broadly accepted in cases of advanced thyroid cancer (Pozdeyev et al., 2015).

### 4.5. Pancreatic cancer

In a study conducted, a glutathione (GSH)-sensitive micelle (PAH-SS-PLGA) was formulated, which consisted of alpha-tocopheryl succinate (TOS) and curcumin, to target pancreatic cancer to provide combinational delivery. The objective was to improve the stability, solubility, bioavailability, and therapeutic efficacy. The optimized formulation of TOS or curcumin-loaded micelles was in the size range of 172.93 ± 1.1 nm and 194.17 ± 1.7 nm, respectively, and the encapsulation efficiency of the PAH-SS-PLGA micelle was reported to be 85% for TOS and 95.5% for curcumin. The results for *in vitro* cytotoxicity assays, using PAN02 pancreatic cancer cells, exhibited higher toxicity for nanoformulated TOS and/or curcumin as compared to free TOS and/or curcumin. Furthermore, it was confirmed that nanoformulated TOS/or curcumin than free TOS/or curcumin at a lower dosage showed better antiproliferative potential for the long term. The calculated CI values were observed to be less than one, which indicates a stronger synergistic effect for the nanoformulated drugs (PAH-SS-PLGA-TOS-curcumin) on cellular proliferation. Thus, it can be concluded that the selected drugs in combination can pave the way for enhancing the therapeutic efficacy and act as a treatment option for pancreatic cancer (Debele et al., 2020).

### 4.6. Lung cancer

Cisplatin, a platinum-containing compound, is involved with the cytotoxic effects in cancer. Epigallocatechin (EGCG), a green tea derivative, has been reported to enhance chemotherapy and target therapy in lung cancer cells. Hence, a recent study performed by Ju Chen and associates, introduced a dual drug delivery system, that involved the formulation of EGCG/cisplatin-loaded gelatin NPs (GE-Pt NPs) to analyze their suppressing power in proliferating lung cancer cells and test their synergistic antitumor capacity. In this case, optimized GE-NPs possessed size diameter of 74.4 ± 9.7 nm and zeta potential of +19.38 ± 0.25 mV. The encapsulation efficiency of cisplatin in GE-Pt NPs was about 63.7% and the EGCG loading rate was around 89%. Higher cytotoxicity levels were observed in A549 cells at low drug concentrations of cisplatin at 2 μg/mL and EGCG at 5 μg/mL. At last, the researchers concluded that a combination approach of EGCG and cisplatin in NPs would be a promising anticarcinogenic candidate for treatment of lung cancer (Chen et al., 2020).

Research was done to formulate PTX and triptolide-coloaded lipid polymer NPs (P/T-LPNs) to evaluate the therapeutic potential of the combination in human lung cancer cells, as a result of synergistic effect that offers reduced drug resistance. The characterization results reported that particle size was around 160 nm, PDI was observed to be less than 0.2 and zeta potential about −30 mV. Additionally, the entrapment efficiency of PTX and triptolide loaded in lipid-polymer hybrid NPs (LPNs) was over 85%, while the drug loading values calculated for PTX and triptolide were approximately 10% and 6%, respectively. Significant cytotoxicity results were obtained with dual drug-loaded LPNs as compared to single drug loaded LPNs. With the use of combination approach, it was found that a synergistic effect was seen when the PTX:triptolide weight ratio was 5:3, which indicates it as a suitable combination. P/T-LPNs strongly inhibited tumor growth, which further proves the anticarcinogenic activity of the two drugs coloaded in LPNs, which can act as a therapeutic option for treatment of lung cancer (Liu et al., 2018).

The study was conducted, which analyzed a combination strategy, involving DOX and Hsp90 inhibitor ganetespib (GT), as a therapeutic alternative to combat non-small-cell lung cancer (NSCLC). DOX was selected as it initiates DNA damage and plays a key role in generation of reactive oxygen species (ROS) via redox-cycling and was combined with second-generation Hsp90 inhibitor, to exert a synergistic effect. As observed by encapsulation studies, enhanced stability, higher therapeutic efficacy, and reduced systemic toxicity, the resulting formulation showed higher drug payload. The combined drugs led to an active destruction of about 80% of lung cancer cells within 48 hours of incubation. The therapeutic effect of DOX was upgraded with the use of GT, through ROS production, which diminishes the cardiotoxicity levels of DOX to a little extent. It was clearly evident from the apoptosis and necrosis assays that GT had a synergistic effect on DOX’s activity. Therefore, the study came to a conclusion that offering such a combinational treatment of DOX and GT would be appropriate for targeting K-RAS driven NSCLC (Sulthana et al., 2017).

In one of the research, CD133+ specific peptide TISWPPR (TR) modified NLC (T-NLC) were prepared in order to target cancer cells along with cancer stem cells (CSCs) simultaneously as an effective therapeutic approach. The combination of PTX and Sal was utilized to achieve the desired response. The optimized formulation exhibited the size range of 128.73 ± 2.09 nm and zeta potential was observed to be −28.3 ± 0.4 mv. The TR-PEG-modified Sal loaded NLC (T-S-NLC) showed entrapment efficiency and drug loading values as 95.62 ± 1.46% and 1.02 ± 0.06%, respectively. The *in vitro* targeting effect revealed the capability of TR modified NLC in enhancing the drug internalization efficiency of CD133 + CSCs. The *in vitro* targeting assay in CD133 + CSCs revealed effective internalization efficiency. The proliferation inhibition action and cellular uptake in NCI-H1299 and S180 cell lines observed was successful in showing the *in vitro* tumor targeting effect of T-S-NLC + small peptide AEYLR-PEG-NLC. While examining *in vivo* tumor targeting action, a significant tumor accumulation was observed (Zhou et al., 2019).

### 4.7. Gastric cancer

Yang and coworkers synthesized hyaluronic acid (HA) containing lipid NPs coloaded with cisplatin plus SOR, using PEG as a mediator for the treatment of gastric cancer. The optimized formulation of HA-PEG-cisplatin and SOR coloaded NPs (H-CS-NPs) had a particle size of 173.2 ± 5.9 nm and zeta potential of −21.5 ± 3.2 mV. *In vitro* cytotoxicity of drug-loaded NPs and free drugs was tested on two types of gastric cancer cell lines: MKN28 and SGC7901 cells. H-CS-NPs effectively inhibited the tumor cell viability of cancer cell lines in contrast to the free drugs. The results interpreted by *in vivo* studies showed that NPs inhibited the tumor volume efficiently, which is attributed to the synergistic efficacy of two drugs (Yang et al., 2018).

### 4.8. Prostate cancer

Curcumin, a compound involved with obstruction in the cell proliferation along with metformin, which acts as a promising candidate in exhibiting anticancerous response were selected to explore their synergistic outcome on lymph node carcinoma of the prostate (LNCaP) cancer cell line. The data with respect to the IC_50_ value, were reported to be 25.01 and 18.66 µM for 24 and 48 hours, respectively, whereas 9.904 and 6.652 mM, respectively, for 24 and 48 hours. Referring to the gene expression, Bax gene was associated with upregulation, as compared to the Bcl-2 gene, which underwent downregulation, as a result of the metformin treatment. A remarkable rise by 0.47- and 0.53-fold in PUMA gene expression was noted due to the treatment received by metformin and the combination, while expression of telomerase reverse transcriptase (hTERT), mammalian target of rapamycin (mTOR), and p53 experienced a huge decline. Moreover, an elevated apoptosis rate (*p* < .05) was achieved (about 24%) with the combination therapy and a successful synergistic potential was proved within a time period of 24 hours. The findings led to a conclusion that in contrast to individual, combined therapeutic treatments offer enhanced efficacy for prostate cancer (Eslami et al., 2020).

Inhibiting COX expression and identifying glut-1 receptors could be an efficient strategy to provide a suitable treatment option for prostate cancer. Considering these factors, a recent study designed a liposomal nanocarrier, encapsulated with celecoxib and genistein, due to their antitumoral properties. The antiproliferative effects of single drugs induced around 20% reduction in cell viability of PC-3 cells, while approximately 75% decrease was seen in the case of combined liposomal formulation, which embarks on the combined capacity of drugs. The wound healing assay depicted a short duration therapeutic effect of celecoxib and genistein for over 24 hours, which was further diminished after prospective exposure, as opposed to their combination, which aids in long-term therapeutic benefits in inhibiting PC-3 cells. Reactive oxygen species were not formed when cells were treated with empty liposomes (EL), celecoxib liposomes (CL) and genistein (GL), while a 3-fold rise in production of ROS was obtained in the cells subjected to celecoxib-genistein complex treatment. Thus, the employed synergistic mechanism of the combined drugs acts in multiple ways to fight prostate cancer cells (Tian et al., 2019).

The combined mechanisms of chemo and gene therapy are proposed for improving the therapeutic efficiency of docetaxel. Utilizing this approach, Bulmahn and coworkers, synthesized siRNA conjugated docetaxel and interleukin-8 nanocarriers, coated with PLGA and chitosan to form lanthanide-doped upconversion NPs. There was a remarkable reduction (75%) in the genetic expression of interleukin-8, when loaded with a dose of upconversion NPs (UCNP) attached siRNA conjugate. This outcome confirms the gene silencing process, induced by the nanocomplex and further exploits its use as a geno-therapeutic candidate to act as an adjuvant to docetaxel for castration resistant prostate cancer. 52% release was observed for docetaxel in phosphate buffer, while a 62% release was exhibited in acetate buffer medium. This depicts a dependency on the pH conditions for the nanocomplex to release the drug. The reduction in IC_50_ values along with the inclusion of siRNA highlights the combined efficacy of IL-8 linked siRNA to enhance the effect of docetaxel. The results demonstrate reliable results of the action of docetaxel against prostate cancer (Bulmahn et al., 2020).

### 4.9. Ovarian cancer

Therapeutic strategies assisted by NPs are considered as an efficient approach for improved anticancerous response. One such instance, employed the combination of cisplatin and PTX and encapsulated them into a three-layered telodendrimer as an effort to minimize the toxic effects and enhance their combined targeting potential for ovarian cancer. The micelles were reported to have a critical micellization concentration (CMC) value of about 29.6 µg/mL. Telodendrimer micelles (TM) exhibited different zeta potential, i.e., about −11.3 mV in pure water, whereas about 3.1 mV after cisplatin complexation. The size determined for optimized micelle was observed to be 9.0 ± 2.6 nm. In addition to the above, the loading efficiency and content of the drugs were calculated as 10% and 97%, respectively. PTX loaded telodendrimer micelles (TM) showed a faster release rate in contrast to cisplatin loaded TM. When referring to the *in vitro* assay using SKOV3 ovarian cancer cells, the most promising antitumor effect was achieved at a combined ratio of 2:1 for cisplatin/PTX. A significant tumor inhibition is attributed to the synergistic combination of cisplatin and PTX. Hence, the proposed combination offering multitude of advantages can be targeted as an effective tool to fight ovarian cancer (Cai et al., 2015).

The combination of cisplatin and PTX was selected for encapsulating into folate decorated nanogels, as a measure to achieve desired therapeutic potential and minimize toxicity related episodes. The optimized formulation represented size of about 90 nm and zeta potential value as −20 mV. A two-fold increase in cytotoxicity levels was observed when folate receptor + A2780 cells were treated with folic acid (FA)-(cisplatin + PTX)/nanogel in contrast to (cisplatinn + PTX)/nanogel. Synergistic activity of the combination exhibited a major cytotoxic response, besides aiding in the tumor suppression. Moreover, IP administration of cisplatin and PTX, was successful in producing decreased serum CA-125 levels. A significantly improved tumor inhibition effect was obtained in murine model of folate receptor-positive ovarian cancer, thus proving it an effective candidate for ovarian cancer (Desale et al., 2015).

### 4.10. Cervical cancer

Chemotherapeutic strategies in combinatorial form have a suppressive effect on cancerous cells. In a recent study performed by Yuan and Gurunathan, a synergistic combination was developed by incorporating graphene oxide and cisplatin into the matrix of silver NPs. WST-8 method led to the determination of combined potential of the conjugate, which employed cisplatin and oxiplatin as the precursors. The increase in cellular growth was observed with rising amount of drug moieties, whereas the inhibitory concentration differed which was observed as 10.0 µm and 12.5 µm for cisplatin and oxiplatin, respectively. A notable rise in hampered cellular growth was reported with increasing concentration of cisplatin and conjugate synthesized. About 25% reduction in cells occurred due to the dose of cisplatin, while the prepared complex induced 30% decline in cell viability, as compared to the cells untreated. This emphasizes on the synergistic mechanism provided by the individual moieties and resultant chemotherapeutic action achieved (Yuan & Gurunathan, 2017).

Wang investigated the synergistic capability of carboplatin and PTX formulated into lipidic nanocarriers, linked to FA in HeLa cell lines. The optimized formulation loaded with FA exhibited 170 nm particle size, whereas the unloaded one had a diameter of 121.3 nm. Carboplatin and PTX conjugated NPs showed no difference in the release profile of the drugs at variable pH environment, but folic acid complex showed elevated response in acidic conditions, compared to basic. Effective internalization of the optimized FA-NPs, observed in HeLa cells is an indicator of endocytosis and macropinocytosis. The nanocomplex was successfully localized within the tumor sites, which is attributed to the EPR mechanism. The formulation showed notable antiproliferative ability, thereby acting as a reliable candidate for cervical cancer (Wang, 2020).

In a study conducted by Murugesan et al., constituent of curcumin, ST06 was loaded into the surface of silver NPs in order to investigate the synergistic tumor inhibiting mechanism. The diametric size of NPs was found to be between 73.48 to 74.52 nm, while the PDI and zeta potential were calculated as 0.2 and −35.3 mV, respectively. The inhibition rate in HeLa cell line was enhanced with increase in dosage of formulation. There was a decline in EAC tumor volumetric values on administration of optimized NPs, in contrast to the control. Aspartate transaminase (AST) and alanine transaminase (ALT) levels were not affected by the formulation, whereas urea levels were elevated following treatment. The results are representative of the antitumor efficacy and antiproliferative response against Ehrlich’s ascites carcinoma (EAC) tumors and HeLa cells (Murugesan et al., 2019) (Table 2).

**Table 2.** Onconanotherapeutics for the management and treatment of cancer.

S. No	Drug 1	Drug 2	Type of nanocarriers	Type of cancer	Types of study	Reference
**1.**	Gemcitabine	Isocombretastatin A-4(isoCA-4)	Nanocomposites	Colon cancer	*In vitro*	(Maksimenko et al., 2014)
**2.**	PTX	Gemicitabine	N-succinyl chitosan NPs	Colon cancer	*In vitro/ in vivo*	(Guo et al., 2015)
**3.**	5-FU	BEZ-235 (Akt) inhibitor	NPs of layered double hydroxide	Colon cancer	*In vitro*	(Chen et al., 2014)
**4.**	5-FU	Curcumin	Chitosan NPs	Colon cancer	*In vitro*	(Anitha et al., 2014)
**5.**	5-FU	Cytotoxic suicide gene E	Poly(ε-caprolactone) NPs	Colon cancer	*In vitro*	(Ortiz et al., 2012)
**6.**	DOX	PTX	Solid lipid nanoparticles	Colorectal cancer	*In vitro*	(Serpe et al., 2004)
**7.**	PTX	BEZ235	Nanoemulsion	Colon cancer	*In vitro/ in vivo*	(Hu et al., 2021)
**8.**	Diallyl trisulfide	DOX	Lipid-based NPs	Colorectal cancer	*In vitro/ in vivo*	(Alrumaihi et al., 2022a)
**9.**	Diallyl Disulfide	Oxaliplatin	Liposome	Colorectal cancer	*In vitro/ in vivo*	(Alrumaihi et al., 2022b)
**10.**	DOX	Curcumin	pH-sensitive core-shell NPs	Glioma	*In vitro/ in vivo*	(Xu et al., 2018)
**11.**	DOX	Lapatinib	Polymeric NPs	Breast cancer	*In vitro*	(Guo et al., 2020)
**12.**	Cisplatin	Gemcitabine	PLGA-NP	Bladder cancer	*In vitro/ in vivo*	(Miao et al., 2014)
**13.**	Rapamycin	Cisplatin	PLGA-NP	Melanoma	*In vitro/ in vivo*	(Guo et al., 2014)
**14.**	DOX	Mitomycin C	Polymeric conjugate NPs	Hepatoma	*In vitro/ in vivo*	(Luo et al., 2015)
**15.**	DOX	Verapamil	Hollow mesoporous silica NPs	Multidrug resistance tumor	*In vitro*	(Palanikumar et al., 2017)
**16.**	DOX	Docetaxel	Janus NPs	Liver cancer	*In vitro/ in vivo*	(Zhang et al., 2018)
**17.**	β-Lapachone	DOX	Polymeric NPs	Multidrug resistance tumor	*In vitro/ in vivo*	(Ye et al., 2017)
**18.**	β-Lapachone	PTX	Prodrug-based NPs	Breast cancer	*In vitro/ in vivo*	(Wang et al., 2019)
**19.**	PTX	Celecoxib	Surface charge-switchable nanosphere	Fibrosarcoma	*In vitro/ in vivo*	(Huang et al., 2019)
**20.**	PTX	Sunitinib	Polymeric micelle	Solid tumor	*In vitro/ in vivo*	(He et al., 2019)
**21.**	DOX	Combretastatin A4	Polypeptide Nanocarriers	Breast cancer	*In vitro/ in vivo*	(Dong et al., 2015)
**22.**	Sal	PTX	Prodrug NPs	Cancer stem cells	*In vitro*	(Liang et al., 2018)
**23.**	Sal	PTX	Polymeric NPs	Breast cancer	*In vitro*	(Muntimadugu et al., 2016)
**24.**	DOX	Curcumin	Lipid-coated polymeric NP	Osteosarcoma	*In vitro/ in vivo*	(Wang et al., 2016)
**25.**	Borneol	DOX	Dendrimers	Brain tumor	*In vitro*	(Han et al., 2018)
**26.**	Oxaliplatin prodrug	PEGylated photosensitizer	PEGylated nanocarriers	Colon cancer	*In vitro/ in vivo*	(Deng et al., 2021)
**27.**	DOX	Astragaloside IV	Liposomes c	Breast cancer	*In vitro/ in vivo*	(Ghosh et al., 2021)
**28.**	Embelin	TRAIL plasmid	pH-sensitive amphiphilic polymeric NPs	Breast cancer	*In vitro*	(Ghosh et al., 2021)
**29.**	Gemcitabine	Docetaxel	Liposome nanocomplexes	Breast cancer	*In vitro/ in vivo*	(Ghosh et al., 2021)
**30.**	DOX	Celecoxib	Glycol chitosan pH-sensitive NPs	Breast cancer	*In vitro/ in vivo*	(Ghosh et al., 2021)
**31.**	Vinorelbine	Tetrandrine	Liposomes with modified PEG conjugates	Brain glioma	*In vitro/in vivo*	(X.-T. Li et al., 2016)
**32.**	Vinorelbine	Tetrandrine	Liposomes modified with RGD (arginine glycine aspartic acid) tripeptide	Brain glioma	*In vivo*	(Li et al., 2019)
**33.**	Daunorubicin	Honokio	Lactoferrin (Lf)-modified liposome	Brain glioma	*In vitro/ in vivo*	(Liu et al., 2017)
**34.**	Transferrin-modified vincristine	Tetrandrine	Liposomes	Brain glioma	*In vitro/ in vivo*	(Song et al., 2017)
**35.**	Cisplatin þ	DOX + camptothecin	Polymeric NPs	Breast cancer	*In vitro/ in vivo*	(Deng et al., 2014)
**36.**	siRNA	Cisplatin	PLGA NPs	Prostate cancer	*In vitro/ in vivo*	(Xu et al., 2013)
**37.**	miR-200c	Docetaxel	PEG-PEP-PCL NPs	Gastric cancer	*In vitro/ in vivo*	(Liu et al., 2013)
**38.**	PTX	Lonidamine	mPEGeb-PCLeb-PPEEA NPs	Ovarian cancer	*In vitro*	(Milane et al., 2011a).
**39.**	Plk1-specific siRNA	PTX	mPEGeb-PCLeb-PPEEA NPs	Breast Cancer	*In vitro/ in vivo*	(Sun et al., 2011)
**40.**	Combretastatin A4	PTX	Polymeric NPs	Lung cancer	*In vitro/ in vivo*	(Wang & Ho, 2010)
**41.**	Doxorubicin	Loperamide	PLGA NPs	Glioblastoma	*In vivo*	(Gelperina et al., 2010)
**42.**	Cisplatin	Docetaxel	PLGA-PLA NPs	Prostate cancer	*In vitro*	(Kolishetti et al., 2010)
**43.**	DOX	P-glycoprotein siRNA	Silica NPs	Cervical tumor	*In vitro*	(Meng et al., 2010)
**44.**	PTX	Alendronate	Dendrimers	Bone metastases	*In vitro*	(Clementi et al., 2011)
**45.**	Cisplatin	Docetaxel	Dendrimers	Prostate cancer	*In vitro*	(Kolishetti et al., 2010)
**46.**	PTX	Thymoquinone	Dendrimers	Breast cancer	*In vitro*	(Soni et al., 2015)
**47.**	Ionidamine	PTX	Dendrimers	Breast cancer	*In vivo*	(Milane et al., 2011b)
**48.**	Cisplatin	PTX	Dendrimers	Ovarian cancer	*In vitro/ in vivo*	(Desale et al., 2013)
**49.**	Docetaxel	DOX	Dendrimers	Prostate carcinoma	*In vitro/ in vivo*	(Wang & Ho, 2010)
**50.**	Verapamil	Vincristine	Dendrimers	Breast carcinoma	*NA*	(Song et al., 2009)
**51.**	PTX	Gemcitabine	Dendrimers	Pancreatic	*In vitro*	(Aryal et al., 2010)
**52.**	DOX	PTX	Carbon nanotubes	Head-neck cancer	*In vitro*	(Colley et al., 2014)
**53.**	DOX	Curcumin	Carbon nanotubes	Hepatocellular carcinoma	*In vitro*	(Anajafi et al., 2017)
**54.**	Camptothecin	DOX	Carbon nanotubes	Head-neck cancer	*In vitro*	(Thambi et al., 2012)

PTX: Paclitaxel; 5-FU: 5-Florouracil; Sal: Salinomycin; DOX: Doxorubicin.

## 5. Discussion

Anticancer chemotherapy, radiotherapy, and surgery have played a pivotal role in managing and treating the different types of cancers. However, these therapeutic approaches have failed to achieve significant clinical outcomes, and relapse and side effects were commonly reported (Conlon et al., 2019). Therefore, the combined drug delivery system is gaining popularity to overcome the shortcomings of single drug delivery and for better therapeutic outcomes. Thus, attempts were made to combine as chemotherapeutic with chemotherapeutic, chemotherapeutic with herbal drug, herbal drug with herbal drug, chemotherapeutic with immunotherapeutic drug for the depletion of tumor cells and to, prevent tumor relapse, and ultimately increase the clinical response (Da Silva et al., 2016). When these combination approaches are being used, pharmacokinetic as well as pharmacodynamic synergistic effect are observed. It is further important to understand that, nowadays, chemotherapeutic and immunotherapeutic drugs are gaining more attention because this combination effectively reduces the level of cancerous T regulatory cells and creates an immune-rich and favorable environment for the anticancer effect of drugs. Clinical translation of preclinical study determines the success or rationale of the research. Considering the nano-oncotherapeutics, CYT-6091 which consists of recombinant TNF attached with the PEGylated, 27was the first nano formulation that was evaluated among patients of advanced solid tumor (NCT00356980, NCT00436410). The same formulation has been successfully studied against breast cancer. Apart from the CYT-6091, AuroLase is the FDA-approved nanoformulation and has been studied among the patients of lung tumor and head-neck carcinoma (Beik et al., 2019).

Hence, looking into the risk-benefit ratio of drugs combination of chemotherapy and immunotherapy appears to be more rational and effective, but at the same time, drug combination increases the cost of treatment frequency of dosing and exhibits serious side effects (Yasmeen et al., 2021). Hence, to overcome such an issue, low-dose immunotherapeutic or chemotherapeutic drugs were initially used, but the pharmacokinetic limitation was still prevalent (Da Silva et al., 2016).

Hence, to overcome these limitations, nanocarrier-based drug delivery is used to improve the pharmacological effect and to reduce the pharmacokinetic limitation and side effects (Correia et al., 2021). Furthermore, the combinatorial nanocarrier system is being used in the treatment of cancer to achieve precise therapeutic value through dual drug delivery. As discussed above, there are a variety of combination approaches have been reported for treatment of different cancers, such as colon cancer, colorectal cancer, breast cancer, bladder cancer, melanoma, brain tumor, lung cancer, etc. Combinatorial nanocarrier-based drug delivery offers certain benefits, such as rationale integration of drugs that cumulatively deliver the drug at the target site with a distinct selection of normal and cancerous cells (Wang & Huang, 2020). The clinical importance of nanocarrier-based chemotherapeutic drug delivery is evident from the studies where gold NPs are under clinical trial for advanced solid tumors (NCT00356980, NCT00436410**)**. Moreover, for the treatment of ductal carcinoma and breast cancer, a gold NP ‘AuroLase’ has been approved by the USFDA (NCT00848042, NCT01679470) (Beik et al., 2019).

## 6. Future prospect and conclusion

No doubt, currently number of anticancer drugs are used as nanomedicine and monotherapy with a targeted therapy approach. However, despite being their wide spread use, these agents failed to exhibit complete remission. Hence, attempts were made use combination of nanomedicine that exhibit better clinical outcome and overcome the possible drug resistance. Despite being a potent and futuristic therapeutic option, nanocarriers for the dual delivery of anticancer drug development is a challenging task and prediction for the reduction in toxicity and targeted drug delivery at a predetermined rate is necessary. Furthermore, it is also crucial to predict, determine and validate how the combinatorial nanocarriers will behave in the tumor microenvironment. Hence, it can be concluded that combinatorial onconanotherapeutics would be an emerging and clinically relevant therapeutic modality for managing and treating cancer. There also exists an unmet and urgent need for the rationale development of a combinatorial onconanotherapeutics system that can be taken from the preclinical to the clinical phase of trials so that more and more chemotherapeutically treated patients can be benefited from this novel treatment approach. Additionally, a well-designed and fabricated nanocarrier can be used for effective gene delivery. In the coming days, this approach will be a more viable and versatile strategy for improving patients’ clinical outcomes and quality of life. Furthermore, it has also been concluded that combinatorial nanocarriers could be a way toward the development of personalized theranostic medicine where different nanocarriers loaded with anticancer drugs can be rationally designed by integrating the expertise of pharmaceutical engineers and biochemists’ oncologists, and clinicians. However, a major limitation for the clinical use of these combinational nanomedicine is the toxicity and hence, these drugs must be fabricated in such a way that they can exhibit multifactorial mechanism of action with minimum toxicity. Thus, combinatorial nanocarrier-based theranostic medicine in the coming time may transform and revolutionize the management and treatment of cancer.
